# Loss of *epe1 ^+^* extends chronological lifespan in *Schizosaccharomyces pombe*

**DOI:** 10.17912/micropub.biology.001507

**Published:** 2025-02-27

**Authors:** Sohini Basu, Yongqi Xu, Tommy Vo

**Affiliations:** 1 Biochemistry and Molecular Biology, Michigan State University, East Lansing, Michigan, United States

## Abstract

Aging is a complex phenomenon that is characterized by the altered regulation of various biological processes over time. One of these, epigenetics, play a crucial role throughout the different stages of eukaryotic life and its alteration is considered a key molecular hallmark of aging. However, the epigenetic factors which are important for lifespan control remain elusive. Here, we used *S. pombe* as a model organism to study the epigenetic basis of aging. Our study reveals that loss of the *epe1* + gene, encoding for the JmjC domain protein Epe1 , extends chronological lifespan and increases H3K9me3 in aged *S. pombe* cells *.*

**Figure 1. Loss of Epe1 enhances chronological lifespan and H3K9me3 levels during aging.**
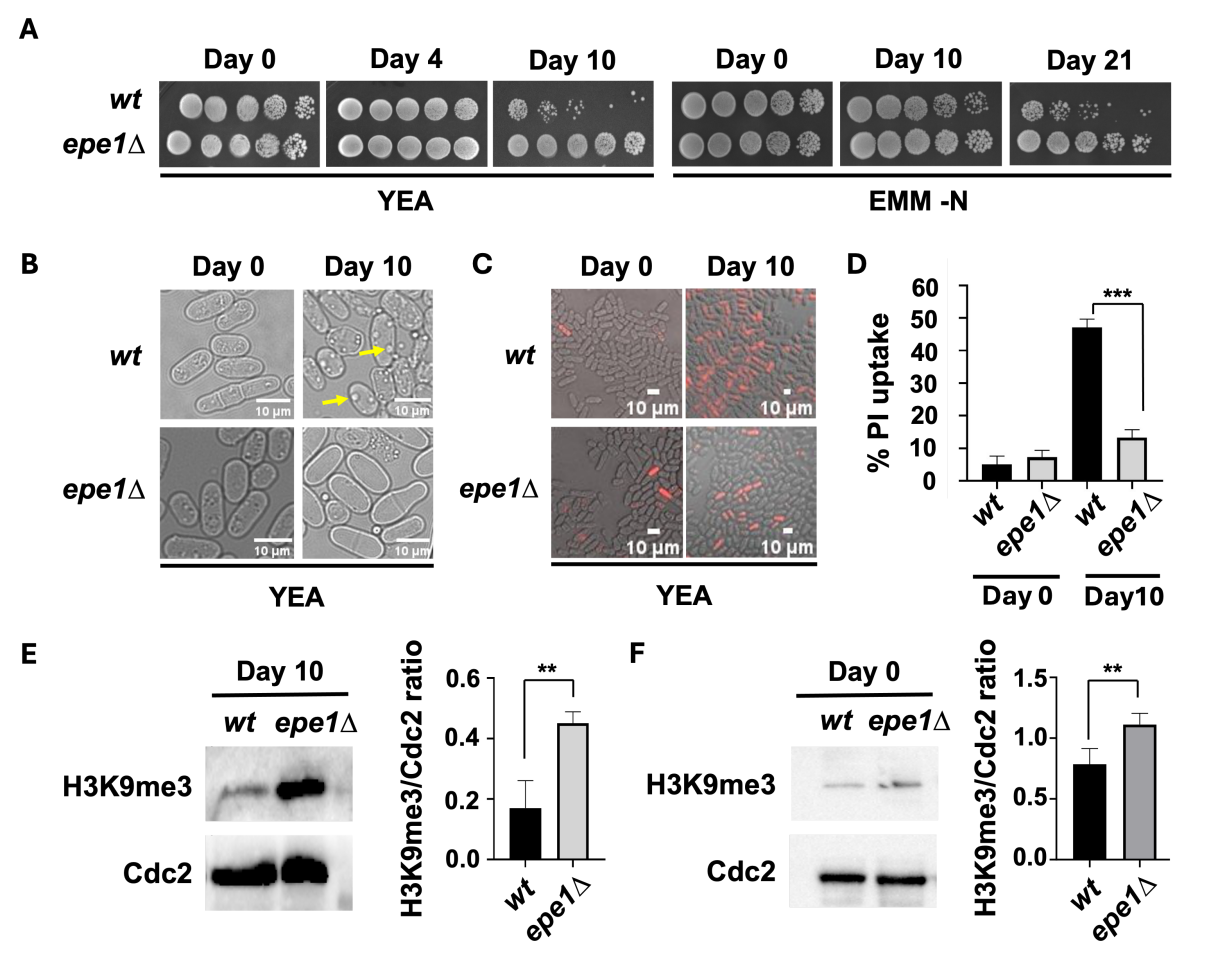
(A) Yeast strains were grown in YEA and EMM minus nitrogen media. At different timepoints they were 4-fold serially diluted and spotted onto YEA medium agar plates and grown for 3-4 days at 32°C. (B) Brightfield images of cells grown in YEA media at Days 0 and 10 was captured at 100X magnification. Yellow arrows point to enlarged vacuole-like structures. Scale bars are shown. (C) Representative fluorescence images of wildtype and *epe1Δ* cells were imaged using an OLYMPUS-DP30BW microscope at 40X resolution at the designated timepoints. Cells were stained with propidium iodide, then washed with phosphate buffered saline prior to imaging. Scale bars are depicted. (D) Percentage of stained cells relative to total cells are plotted. At least 100 individual cells were counted per sample. ***p < 0.001. All p values were obtained from two-tailed unpaired Student's t test. (E) Age 10 cultures were subjected to western blot analysis where total protein was isolated using TCA lysis method of the indicated strains. They were immunoblotted with anti-H3K9me3 and control anti-Cdc2 antibodies. The ratios of the H3K9me3 and Cdc2 western blot signals were plotted. Signal measurements from three independent western blot experiments were taken to calculate the ratios. **p < 0.01. All p values were obtained from two-tailed unpaired Student's t test. (F) Age 0 cultures were subjected to H3K9me3 western blot analyses as described above for panel E. **p < 0.01. All p values were obtained from two-tailed unpaired Student's t test.

## Description

Epigenetics refer to chromatin modifications and small RNAs that regulate gene expression levels without altering the DNA sequence itself (Goldberg *et al.* 2007). In animals, epigenetic alterations involving DNA methylation and tri-methylation of histone H3 at lysine-9 (H3K9me3) are prevalent features of aging (Horvath 2013; Zhang *et al.* 2015) and are thought to be key modulators of the aging process (Pal and Tyler 2016) . These chromatin methylations demarcate heterochromatin, which are regions of the genome where genes are silenced or lowly expressed (Stancheva 2005; Grewal 2023) . Defects in H3K9me3-marked heterochromatin can lead to premature aging disorders including Werner's Syndrome (Zhang *et al.* 2015; Mrabti *et al.* 2024). Identifying factors which regulate aging-associated epigenetics will be important for predicting, diagnosing, and/or treating age-related ailments. To investigate the fundamental molecular basis behind aging, chronological aging (CLS) in yeast is a well-established model system to identify conserved factors that affect cellular mortality (Fabrizio *et al.* 2001). CLS is a measure of how long a cell maintains its viability after it stops dividing at the stationary phase. Although aging in single-celled yeast and multicellular animals differ in many ways (Laun *et al.* 2006), easily manipulable yeast models can provide genetic and molecular clues into how conserved processes that define as aging hallmarks, including epigenetics, rewire over time.

The fission yeast *Schizosaccharomyces* *pombe* has been extensively used to study CLS (Ohtsuka *et al.* 2021). So far, over 80 genes have been reported to regulate *S. pombe* CLS (Ohtsuka *et al.* 2021) . Here, we add to this list by identifying the *epe1* + gene as a novel negative regulator of *S. pombe* CLS. *Epe1* encodes for the JmjC domain protein Epe1 which limits heterochromatin maintenance and spreading by antagonizing H3K9me3 (Zofall and Grewal 2006; Isaac *et al.* 2007). It is known that the loss of Epe1 promotes progressive rewiring of the H3K9me3 epigenetic landscape to enhance adaptation in the presence of short-term stress (Wang *et al.* 2015; Larkin *et al.* 2024). To examine the role of Epe1 in longer-term CLS, we continuously cultured wild-type ( *wt* ) cells or cells lacking *epe1 ^+^* ( *epe1Δ* ) in yeast extract (YEA) liquid media for several weeks at 32°C (standard culturing temperature for *S. pombe* ) with 220 rpm shaking. On Day 0, cells are actively proliferating and are, thus, in exponential growth phase. By Day 1, the cultures have reached maximal optical density (OD _600_ ), have become non-dividing, and have reached stationary phase. At different timepoints, viability was assessed by spotting the aged cells onto nutrient-rich YEA agar plates. At the early timepoints (e.g Day 0), there was high viability as expected ( Figure 1A ). By Day 10, viability was noticeably reduced for *wt* cells but remained higher for *epe1Δ* cells, suggesting that loss of Epe1 extends CLS. To determine whether our finding was specific to the type of culture media, we repeated our CLS experiment using cells that were aged in Edinburgh Minimum Media lacking nitrogen (EMM -N). Similar to aging stationary phased cells in YEA media, cells in EMM -N become non-dividing and can maintain viability potential for some time (Sideri *et al.* 2015). A key difference is that *S. pombe* cells in yeast extract media enter the non-dividing state via G2 phase while those in EMM -N enter via G1 phase (Wei *et al.* 1993). While aged cells in EMM -N displayed longer CLS compared to those that were aged in YEA media, we found that *epe1Δ* cells still had higher CLS compared to *wt* cells ( Figure 1A ). This suggests that *epe1Δ* extends CLS in *S. pombe* cells, regardless of the media choice between YEA and EMM -N. We subsequently focused on cells aged in YEA liquid media, as the shorter CLS in this media type was more suitable for the detailed characterization of *epe1Δ* cells.

We then used light microscopy to observe individual cells at Day 0 and Day 10. While *wt* and *epe1Δ* cells appeared similar at Day 0, we noticed by Day 10 that *wt* cells markedly differed from *epe1Δ* cells based on the appearance of cell shrinkage and apparently increased vacuole size within *wt* cells ( Figure 1B ). These morphological changes are consistent with those that were previously reported for *wt* strain SP14000 that were chronologically aged in yeast extract media (Roux *et al.* 2009). Because these phenotypic alterations in the *wt* cells could be associated with increased cellular stress and/or reduced viability (Keuenhof *et al.* 2022), we assessed cell viability at the single-cell level by fluorescently measuring the uptake of propidium iodide (PI). PI is a dye that stains nuclear DNA only if it can penetrate membranes that typically become porous upon cell stress or death (Deere *et al.* 1998). We observed that, by Day 10, there were more PI-stained *wt* cells compared to *epe1Δ* cells ( Figure 1C ). The difference in number of PI-stained cells between *wt* and *epe1Δ* was statistically significant ( Figure 1D ). Findings from these single-cell analyses are consistent with our population-level observations from our yeast spotting experiments ( Figure 1A ). Finally, we assessed the impact of *epe1Δ* on H3K9me3 levels in Day 10 cells because Epe1 is a known negative regulator of this epigenetic modification (Zofall and Grewal 2006) . By whole-cell western blotting, we found that aged *epe1Δ* cells had elevated global H3K9me3 levels compared to *wt* cells ( Figure 1E ). The elevated H3K9me3 level in *epe1Δ* cells was not specific to Day 10 aged cells because younger (exponentially growing) Day 0 *epe1Δ* cells also showed higher H3K9me3 levels compared to *wt* cells ( Figure 1F ), which is consistent with the known role of Epe1 in promoting H3K9me3 turnover (Aygün *et al.* 2013). These data suggest that Epe1 may promote chronological aging in *S. pombe* by facilitating H3K9me3 loss.

In summary, our study revealed a novel role of Epe1 in CLS regulation. Loss of Epe1 improved long-term cell viability, independent of media choice, and increased H3K9me3 levels in Day 10 aged cells, when compared to *wt* cells. These findings suggest that Epe1 may facilitate the remodeling of H3K9me3-marked heterochromatin in non-dividing aged cells to eventually promote death. The involvement of Epe1 in yeast CLS control opens the possibility that its mammalian homolog(s) could similarly be important for aging processes. *S. pombe* Epe1 is homologous to KDM7A and KDM7B in animals (also named JHDM1D and PHF8, respectively), which are JmjC domain proteins that negatively regulate methylation of H3K9, H3K27, and H4K20 via demethylation (Tsukada *et al.* 2010; Chaturvedi *et al.* 2019). KDM7A/B are implicated in memory and cognition (Wang *et al.* 2023; Fan *et al.* 2024), traits that are aging-associated. However, there is no direct evidence linking these mammalian homologs to aging. Future studies should be aimed to elucidate how Epe1 and KDM7A/B alter the epigenetic landscape during aging.

## Methods

**Yeast culturing and aging**

Yeast strains were cultured in yeast extract media with adenine supplementation (YEA), Edinburgh Minimum Media (EMM), or Edinburgh Minimum Media lacking nitrogen (EMM -N) (Moreno et al., 1991). Cultures were maintained at 32°C, with an approximate culture liquid volume-to-maximum volume (of the culture carrier) at 1:5, and with 220 rpm shaking speed. Aging in YEA cultures was performed by inoculating exponential phased cells into YEA media at a starting OD _600_ of approximately 0.5 and allowing the same culture to continuously shake for various days. For EMM -N aging, exponential phased cells were first grown in EMM, then washed with EMM -N to remove sources of nitrogen from the media, and finally continuously allowed to shake in EMM -N media for various days. *Epe1Δ* cells were constructed using a previously described PCR-based homology-directed gene deletion approach for *S. pombe* (Krawchuk and Wahls 1999) .

**CLS spotting assays**

At each selected time point during the chronological aging process described above, an aliquot of the cultures was transferred into 1.5 mL tubes and normalized by dilution to an OD _600_ of 0.5. Then, 4-fold serial dilutions were performed and 10uL of cells were spotted onto rich YEA agar plates. Plates were incubated for 3–4 days at 32°C prior to being imaged using a BioRad ChemiDoc. Spot assays were performed in triplicate with representative images shown in the figure.

**Microscopy and Image Analysis**

For microscopic analyses, 200uL of cells were collected at designated time points and incubated in the presence of propidium iodide (PI) for 15 mins at room temperature. PI was used at 5 μg/mL working concentration, dissolved in water. Three PBS washes were done and cells were resuspended in 10μL of PBS. Slides were prepared and observed using the 40X objective of an OLYMPUS-DP30BW fluorescence microscope.

**Western Blot**

Cells were subjected to the TCA lysis and protein precipitation method as we previously described (Xie *et al.* 2019). Total protein was resolved using 12% SDS PAGE gels. Blots were probed with anti-H3K9me3 antibody (Active Motif, 39161) at 1:1,000 dilution. Anti- cdc2 (Santa Cruz, sc-53217) was used at 1:2,000 dilution.

## Reagents

**Table:** 

**Strain**	**Strain collection**	**Genotype**
*wt*	TVSB01	*Mat1-Msmt0 leu1-32 ade6-M210 his2 ura4-DS/E otr1R(Sph1)::ura4+*
*epe1Δ*	TVSB02	*Mat1-Msmt0 leu1-32 ade6-M210 his2 ura4-DS/E otr1R(Sph1)::ura4+ epe1Δ::natMX*
